# Iritis

**Published:** 1891-12-12

**Authors:** 


### OPHTHALMOLOGY.

### IRITIS.

Inflammation of the iris may arise from either local or constitutional causes. It may be from direct injury to the iris itself, or to some neighbouring tissue. Though the iris resents injury, it can nevertheless be cut, as in the operation of iridectomy, without any untoward result; it is when it is bruised, torn, or much dragged upon, as when entangled in a wound of the cornea, that it so readily inflames. Iritis may be excited by other local means, viz., tumours of the iris, grummata, tubercles, sarcomata, dermoid, &c.

The constitutional causes of iritis are acquired syphilis, iritis being a common accompaniment of the secondary eruption, in cases which have not had mercurial treatment. Usually one eye is first attacked, the other eye, however, soon participating in the inflammation. In inherited syphilis, Hutchinson observed that the average age for iritis is five months and a half, and that both eyes are attacked simultaneously about as frequently as one alone.

In the rheumatic dyscrasia, a recurrent and very insidious form of iritis occurs. It is not seen in acute rheumatism, but often follows in the wake of prolonged sub-acute attacks of the articular form, in those suffering at intervals from muscular and fascial rheumatism after exposure to damp and cold, and in the victims of gonorrhœal rheumatism; in this latter form it often attacks both eyes at once, but in the other forms one iris is, as a rule, alone affected; and this aids its diagnosis from syphilitic iritis with rheumatoid pains in the limbs, in which the second eye is, as before stated, attacked soon after the first.

It is not unfrequently found in gouty persons, the same recurrent insidious form as in the rheumatic subject.

Pain is an uncertain symptom, being often absent, as is photophobia.

The sight is affected, and the circumcorneal zone of vessels injected, and pressure wfth the finger, from the cornea outwards, fails to empty the vessels radiating from the cornea, and situated beneath the conjunctiva. On closer examination the iris is found to have lost its brilliant, clear appearance, has sometimes changed colour, and is muddy-looking, there being an effusion of lymph, serum, or even blood into its substance, or may be, into the anterior chamber (Hypopion); the pupil is not quite circular, and is sluggish in action. After the application of atropine it dilates irregularly, and in the plastic forms adhesions are found to the lens capsule, and deposits of lymph or pigment on the back of the cornea. When only one eye is affected, comparison with its fellow will greatly magnify these various symptoms. In old standing cases of chronic iritis the adhesions may be so firm as to prevent the iris dilating at all, or so to limit it, that the pupil takes on various shapes, like the ace of clubs on a card, &c.

Pathologically, iritis is divided into the *serous* and *plastic* forms.

In the *serous* form the aqueous humour becomes turbid from the presence of serum, the tension of the eyeball is increased, and the iris pushed backwards; but adhesions to the lens are rare, or the inflammation may attack the posterior membrane of the cornea as well (Descemet's membrane).

One may describe the anterior chamber as a capsule, the membrane forming which, is attached to, and covers the posterior surface of the cornea, is reflected on to the anterior surface of the iris, round its free margin to its posterior surface, and then on to the anterior surface of the lens capsule, and it is then seen how easily all these surfaces may be involved in an attack of iritis; and this will also account for iritis following a wound of the cornea and lens capsule, where the iris itself is not punctured.

The *plastic* form is the more important on account of its sequelæ, the exudation is very sticky, and adhesions are liable to form between the iris and lens capsule, the whole of the margin of the iris may become adherent to the lens capsule, and occlusion of the pupil be the result. This is the form occurring in syphilis, rheumatism, and gout.

The treatment of iritis must be prompt to prevent these untoward results, and the local treatment must be the first consideration.

Obtain as great amount of dilatation of the pupil as possible, with atropine. A solution of 4 grs. to the ounce may be used at intervals of an hour; one or two drops should be applied until maximum dilatation is obtained, and then repeated every fourth hour, so that any deposit of lymph on, or attachment of the iris to, the lens capsule may be away from the centre, where it would be more obstructive to vision afterwards.

Light should be occluded, as it tends to contract the pupil, as well as act as an irritant.

Tension of the eyeball must be diminished by *paraceutesis* of the anterior chamber.

Paraceutesis of the anterior chamber is performed thus: the eyelids being held open by a speculum, the ball of the eye is held with fixation forceps, and a triangular knife, or any other small knife, introduced through the corneal margin into the anterior chamber, the incision being made by holding the knife so that its blade is at right angles to the surface of the cornea. Having entered the anterior chamber, its direction is altered so that it may travel between the cornea and iris in a plane, parallel to their surfaces, to the centre of the pupil; the knife is then withdrawn, allowing the aqueous to escape with it, the surgeon keeping the knife close to the back of the cornea as he withdraws it, lest the iris should be washed against and injured by its point.

Inflammation and congestion to be diminished by blood letting, three or four leeches being applied to the temple.

Pain is best relieved by heat, a dry flannel, or spongiopi. line held against a jug of boiling water and then applied to the eyelids is the best form; cocain also locally applied is soothing, and morphia may be injected hypodermically, or given by the mouth.

Mercury is to be administered short of salivation, whether or no there be a syphilitic history, and in syphilis must be continued in small doses for at least six months, even though the iritis be cured; the mercury in other cases may be stopped when the adhesions have given way, and iodides, colchicum salicylates, &c., substituted.

Rest for the eyes must be insisted on, for the disease may be aggravated by the use of them. The patient should be confined to a darkened room. When this treatment fails, *iridectomy*, removal of a portion of the iris must be performed, but it is not good practice in traumatic cases. When adhesions remain and interfere with the sight of the eye, *corelysis* or separation of the adhesions without removal of any of the iris may be performed.

In the early stages of traumatic iritis, cold locally applied is an excellent therapeutic agent, but must not be applied in any other form of iritis.

